# Cloning and phylogenetic analysis of N protein gene from Rift Valley Fever Virus (RVFV)

**DOI:** 10.6026/973206300200091

**Published:** 2024-02-29

**Authors:** Ahmed Mohamed Hassan, Arwa Ahmed Zehairy, Al-Judaibi Awatif Abid, Sayed Sartaj Sohrab, Ibraheem Azhar Esam

**Affiliations:** 1 Special Infectious Agents Unit, King Fahd Medical Research Centre, King Abdulaziz University, Jeddah, Saudi Arabia; 2 Department of Biological Sciences, College of Science, University of Jeddah, Jeddah, Saudi Arabia; 3 Department of Medical Laboratory Sciences, Faculty of Applied Medical Sciences, King Abdulaziz University, Jeddah, Saudi Arabia

**Keywords:** Rift Valley fever virus, RVFV, nucleocapsid (N) protein, cloning, sequencing, phylogenetic analysis

## Abstract

Rift Valley Fever (RVF) is a mosquito-borne viral zoonosis caused by RVFV in humans and livestock. Currently, there are no approved vaccines or antiviral therapies available. Additionally, in Saudi Arabia, there is a lack of a routine screening system to monitor RVFV in humans and animals which hinders to design and develop the preventive measures as well as the prediction of future outbreaks and the potential re-emergence of RVFV. Hence, we have performed the cloning, sequencing, and phylogenetic analysis, of nucleocapsid (N) protein gene. The sequence analysis showed high similarities with RVFV isolates reported from humans and animals. The highest similarity (99.5%) was observed with an isolate from Saudi Arabia (KU978775-Human) followed by 99.1% with four RVFV isolates (Human and Bovine) from other locations. A total of 51 nucleotides and 31 amino acid variations were observed throughout the N protein gene sequences. The phylogenetic relationship formed closed clusters with other isolates collected from Saudi Arabia. Thus, we report of the cloning, sequencing, and phylogenetic analysis of the RVFV-N protein gene from Saudi Arabia.

## Background:

Rift Valley Fever Virus (RVFV) is an RNA arbovirus that causes critical infectious disease in humans and a wide range of domestic livestock such as goats, sheep, and cattle in Africa, the Middle East, the Arabian Peninsula, and other non-endemic regions. The causative agent was first identified in Kenya in 1931. RVFV is a member of the Bunyavirales order, Phenuiviridae family, of negative-sense RNA viruses. RVFV contains three genome segments: The large (L) segment encodes the viral RNAdependent RNA polymerase (RdRp), the medium (M) segment encodes the surface glycoproteins Gn and Gc along with nonstructural protein NSm, and the small (S) segment encodes the nucleoprotein N and the non-structural NSs protein ( [1,2, 3,4,5, 6,7,8, 9,10,11, 12,13,14, 15]. This virus is mainly transmitted by the bite of a wide range of mosquitos that belong to one of the six genera Aedes, Anopheles, Mansonia, Culex, Aedeomyia, and Coquillettidia [16]. Moreover, the non-vector transmission route of the virus can occur in humans when they interact directly with contaminated blood, body fluids, or tissues of infected animals without enough precautions during slaughtering, skinning, breeding, milking, consuming raw animal products, and assisting in animal birth, or carrying fetal animal after abortion [17,18]. There is no human-to-human transmission has been reported to date [19]. RVFV transmission can lead to outbreaks of illness in livestock, which can have significant economic impacts on the affected communities. In humans, RVFV illness can cause disease range from mild flu-like symptoms to severe hemorrhagic fever, which can lead to death in severe cases [6,8,20]. RVFV was first detected while investigating an epidemic among sheep in the Rift Valley of Kenya as it was circulating the African continent causing an epizootic epidemic among humans and non-humans until it reached the Arabian Peninsula in 2000 and caused an outbreak in Saudi Arabia and Yemen [7,21]. At least 215 people died in that outbreak, with more than 1,500 people infected with the virus, and major domestic animal loss was documented [22]. Since the outbreak, Saudi Arabian authorities have implemented several preventive programs to limit the incidence of RVFV which include vaccinating livestock against RVFV, Mosquito surveillance through spraying insecticides, and biological control [23]. However, there is no routine screening that has been done recently on those high-risk groups exposed to RVFV such as slaughterhouse workers to confirm that re-emergence of that virus has not occurred. Nevertheless, NASA mapping reports predict the re-emerging of RVFV in several regions including the Middle East, due to changes in perception scale and an increase in vegetative landscapes [24,25,26]. These data raise concerns about the potential return incidence of the disease in different parts of the globe. Given the significant public health and economic impacts of RVFV, there is continuous ongoing research to better understand the virus and develop an effective prevention and control measures [27]. However, it is important to note that prevention measures largely rely on controlling mosquito populations and avoiding contact with infected animals. The vaccines for RVFV are available only for animals, particularly in both endemic and non-endemic areas. Additionally, despite the availability of supportive medication for managing mild cases of RVFV infection, there is currently no specific antiviral treatment and vaccine for humans [28,29]. These limitations highlight the pressing need for regular monitoring of both animals and humans, specifically slaughterhouse workers and locals within the outbreak zone of 2000 and other parts of Saudi Arabia. However, it is important to note that, currently only approved serological diagnostic tools are limited to use in endemic countries and Disease Control and Prevention reference laboratories, with no approved commercial kits available for detecting RVFV in non-endemic regions [30,31]. The diagnosis of RVFV in both human and animal samples is currently being performed by serological and molecular assays [32, 33,34]. Therefore, a highly sensitive and specific serological diagnostic assays such as enzyme-linked immunosorbent assay (ELISA) is urgently required [35, 36]. Therefore, it is of interest to clone, sequence, and make a phylogenetic analysis to identify any possible emergence of new strains so that a routine screening and continuous surveillance system can be designed and developed to prevent future outbreaks in the Kingdom of Saudi Arabia.

## Methodology:

## Sample collection and virus isolation:

The samples were collected by personnel communication and transported in dry ice to the Special Infectious Agents Unit, King Fahd Medical Research Center, King Abdulaziz University, Jeddah, Saudi Arabia. The samples were further analyzed by serological and molecular assay. The positive sample was used to inoculate the Vero cell line for virus growth and multiplication in a tissue culture flask and incubated at 37°C for 1 hr. The virus was isolated from the infected cell supernatant and further confirmed by laboratory tests and used for viral RNA isolation.

## Viral RNA extraction and Realtime-PCR:

The viral RNA was extracted and purified from the infected cell supernatant using an Automated RNA extraction system in the Biosafety level-3 lab by magnetic beads (MagNa pure compact) as per instructions. The eluted and purified RNA was used to detect viruses using the QuantiFast Probe RT-PCR Kit (QIAGEN).

## PCR amplification, cloning, sequencing, and phylogenetic analysis:

The sequence of the RVFV-N gene was selected from the NCBI-GenBank database (www.ncbi.nlm.nih.gov/genbank/) and used for primer design. The purified viral RNA was used for PCR amplification of the Nucleocapsid protein (N) gene using specific primers. The cDNA synthesis and amplification of the RVFV-N gene were carried out using the One-Step RT-PCR Kit- QIAGEN as per the manufacturer's instruction. Briefly, the reaction was completed in total volume of 50 µl. The mixture consisted of 5x One Step RT-PCR buffer, 200 µM dNTP Mix, 2.0 µl One Step RT-PCR enzyme mix, 0.5 µM of forward and reverse primers, 5 µl of the Viral RNA as template, and the final volume was made up to 50 µl by adding double distilled water.

The forward primer sequence is 5'-TCTCGAGTTAGGCTGCTGTCTTGTAGGC-3' and

the reverse primer sequence is 5'-CAAGCTTTAATGGACAACTATCAAGAGCTT-3'

The cycling condition was designed as 50°C for 30 min for cDNA synthesis followed by 1 cycle at 94°C, 3 min for initial denaturation, and then 35 cycles at 94°C for 1 min for denaturation, and 1 min at 52°C for annealing, and 1 min at 72°C for extension and finally 10 min for the final extension as last cycle. The PCR product was visualized on 1% Agaroses gel and stained with ethidium bromide and recorded the gel image under the Gel doc system (IN GENIUS-Syngene Bio Imaging) to observe and confirm the size of the PCR amplified product by using 1kb DNA ladder as scale (Thermo Scientific). The RVFV-N gene was cloned in the pET-28a (+) vector (GenScript). The PCR-amplified product and vector DNA were eluted and purified from the gel using a PCR purification kit (MOLEQULE-ON) following the manufacturer's instructions. The purified DNA was restricted with the selected restriction enzymes XhoI and Hind III (ThermoScientific). The insert and vector DNA were ligated by using the T4 DNA ligase enzyme. Briefly, Insert DNA (100 ng) and Vector DNA (300 ng) were mixed in a PCR tube by adding the 1µl T4 DNA ligase and 10x buffer (2µl) (ThermoScientific), and the final volume was made to 20 µl by adding the double distilled water and finally incubated at 22°C for overnight. The ligated product was used for transformation into competent *E. coli* (DH5α) cells by using the heat shock method. The transformed *E. coli* cells were spread on Luria Broth Agar (LBA) plates with Kanamycin (50 mg/l) and further allowed to grow at 37°C overnight. The grown bacterial colonies were used for confirmation of recombinant clones by using colony PCR. Briefly, the bacterial colonies were grown on antibiotic plates at 37°C overnight. For colony PCR, the scrap of bacterial colonies was used to amplify the RVFV-N gene. The plasmid DNA was purified from overnight grown culture using the Miniprep Kit (MOLEQULE-ON) and the purified plasmid DNA was used for sequencing. Sequencing was performed at our Special Infectious Agents Unit, by following the instructions of the commercial kit BigDye® Terminator v3.1 cycle sequencing kit from applied biosystems using the Sanger dideoxy sequencing method. The specific gene forward and reverse primers were used for sequencing. The sequencing products were purified using the ethanol/EDTA precipitation method and analyzed with the ABI Prism 3500 genetic analyzer (Applied Biosystems) according to the manufacturer's instructions. The resultant raw sequences were assembled and analyzed by the software program BioEdit (v.7.2), and the final sequences were selected for further analysis. The sequence identity matrix of generated sequences was determined with submitted RVFV genome sequences in the NCBI-GenBank database. The generated nucleotide and amino acid sequences were aligned and analyzed using multiple sequence alignment tools in the CLUSTAL-W program (http://www.ebi.ac.uk/clustalw). The compared sequence file was imported into the MEGAX program and the phylogenetic dendrogram was generated from the aligned nucleotide sequences using neighbor-joining and maximum parsimony methods [37].

## Results:

Sample collection and virus isolation: The collected sample was found to be positive for RVFV infection as confirmed by RVFV-specific RT-PCR assay. The positive sample was used for virus inoculation and growth in cells. The inoculated virus grew successfully in the Vero cells. The virus was successfully isolated from the cells and used for RNA isolation and purification.

## Viral RNA extraction and Real-Time-PCR:

The viral RNA was successfully isolated and purified using the QIAamp Viral RNA mini kit (Qiagen) following the manufacturer's instructions. The Real-Time PCR confirmed the presence of the virus by using specific primers.

## PCR amplification, cloning, and sequence analysis:

**Figure 1:**
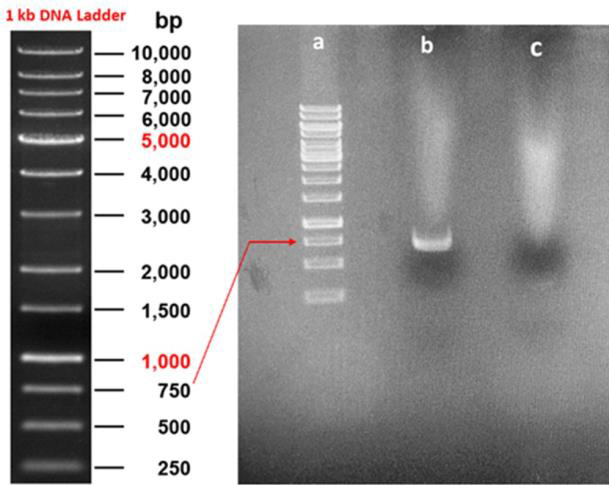
PCR amplification of RVFV-N gene: Lane (a): 1 kb DNA ladder, Lane (b): N gene (750bp), Lane (c): Negative control.

**Figure 2:**
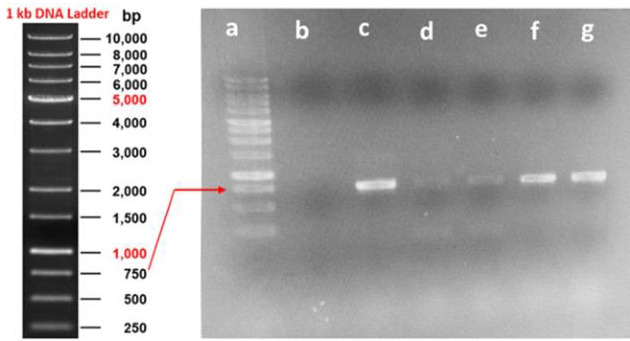
Confirmation of rRVFV-N clones by colony PCR: Lane a: 1kb DNA ladder, b: Negative control, c: Positive control (750bp), Lane d, e, f, g: Confirmed recombinant clones.

**Figure 3:**
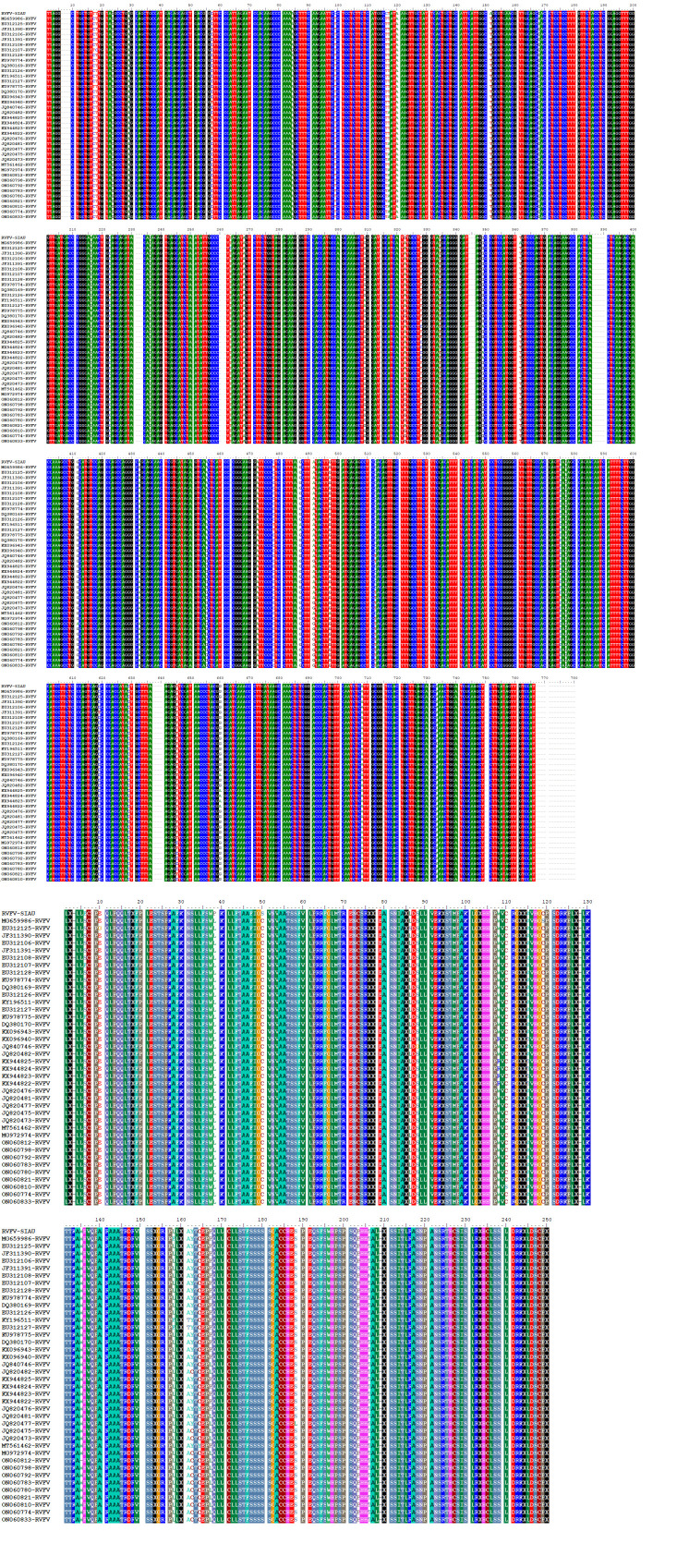
Multiple sequence alignment of RVFV-N gene with selected isolates. (Top) nucleotide variations; (bottom) amino acid variations

The purified RVFV-RNA was used to amplify the RVFV-N gene by Reverse Transcriptase-PCR using the designed specific primers, and an amplicon of ~750 bp was amplified and visualized on 1% Agarose gel (Figure 1). The PCR-amplified RVFV-N gene was purified and cloned into a pET-28a (+) vector. A total of 10 colonies were grown on Luria Britani Agar (LBA) plate with Kanamycin. The putative recombinant colonies (only five) were used for screening by colony PCR. The colony PCR confirmed the amplification of the RVF-N gene (~750 bp) in all five colonies screened (Figure 2). The positive clone was sequenced, and resultant sequences were aligned by BioEdit and initially used for sequence analysis by NCBI-BLAST. Based on the highest homology with the other RVFV sequences submitted to GenBank, we designated our clone as RVFV-SIAU-KSA. The multiple sequence alignment analysis of both nucleotide and amino acids sequences of RVFV-SIAU-KSA showed significant homology with the selected RVFV isolates from various locations and host collected during the years 1976-2020. A total of 51 nucleotides and 31 amino acid variations scattered through the whole RVFV-N gene were observed from the years 1976-2020 (Figure 3a and Figure 3b). The nucleotide sequence identity matrix of RVFV-SIAU-KSA with other RVFV isolates showed the variable sequence identity ranged from 98.5%-99.5% with various strains of RVFV. The highest similarity (99.5%) was observed with Saudi Arabia (KU978775-Human) followed by 99.1% with four RVFV isolates (Human and Bovine) reported from Madagascar and Kenya in 1991 & 1998 collection and the lowest (98.5%) was with many RVFV isolates. The amino acid sequence of the RVFV-SIAU-KSA isolate showed the highest similarity (98.7%) from Saudi Arabia (KU978775-Human) and the lowest identity (96.3%) with two isolates collected from South Africa (EU312127-Bufallo) and Uganda (ON060821-Human) during different periods and hosts. The similarity ranged from 96.7%-97.9% with other RVFV isolates (Table 1).

**Figure 4:**
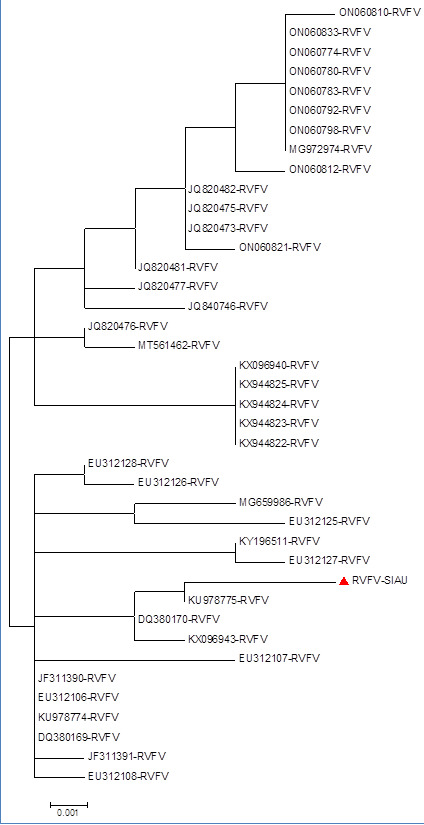
Phylogenetic tree based on nucleotide sequences of RVFV-SIAU-KSA with other isolates.

**Figure 5:**
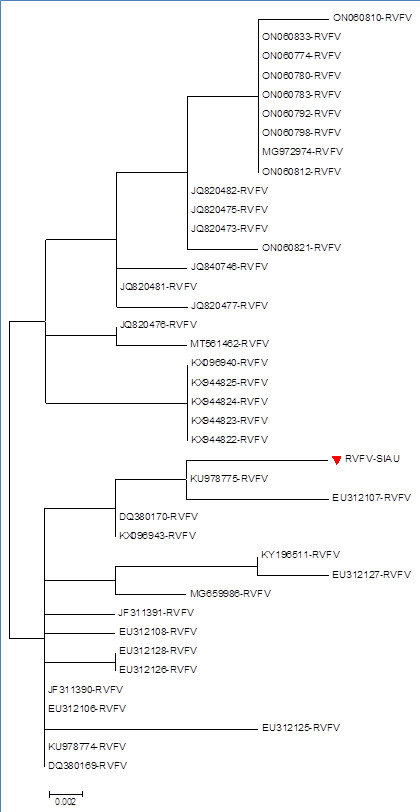
Phylogenetic tree based on amino acid sequences of RVFV-SIAU-KSA with other isolates.

The phylogenetic tree analysis based on the nucleotide sequences showed that RVFV-SIAU-KSA formed a closed cluster with RVFV-Saudi Arabia (KU978775, DQ380170, KX096943), while other isolates formed a separate cluster with different RVFV isolates collected from human and other hosts during multiple collection periods (Figure 4). The amino acids sequences phylogenetic tree of RVFV-SIAU-KSA formed a closed cluster with RVFV (KU978775-KSA-Human and EU312107-Medagasker-Bovine) isolates collected at different collection periods from human and Bovine samples, and remaining isolates from human and other hosts formed a separate cluster. Interestingly, the amino acids phylogenetic tree analysis showed that, only one RVFV isolates (EU312107) from Madagascar-Bovine clustered with all 3 RVFV isolates from KSA. Additionally, a separate clustering was observed among human and other hosts collected during different periods and locations. A mixed clustering was also observed among the various isolates collected during 1976-2020 from different hosts and locations (Figure 5). A similar clustering pattern was observed in both nucleotide and amino acids phylogenetic tree analysis. However, a significant clustering pattern was also observed in both trees with other isolates which indicates that there will be a possibility of the emergence of a new RVFV strain with altered properties in the near future.

## Discussion:

RVF disease was identified for the first time in Kenya in 1931 when the sudden death of livestock was observed on a farm in the Rift Valley region. The causative agent was further identified, characterized, and based on the genomic similarities and other features; it was finally designated as Rift Valley fever Virus (RVFV) [1]. This is a mosquito-borne pathogen that affects both humans and livestock in Africa, Middle East, and other new endemic regions. The virus can be transmitted through direct contact with the infected tissues/samples, handling of sick or deceased livestock, and consumption of raw animal products, mosquito bites, and cause sporadic to widespread morbidity and mortality in domestic livestock as well as humans. The high viremias in infected livestock favoured the legal and illegal trades of livestock globally and due to increased human travel for global trade and commerce as well as the mosquitoes as vector can introduce the RVFV in new areas and environment. This virus can cause large abortion storms with 100% loss of fetuses of pregnant animals while 90-98% of humans have asymptomatic infections which results in a serious impact on the global economy, agriculture, and public health [5,6, 20,38]. This virus is one of the eight viruses and has been listed in the list of blueprints of the World Health Organization [39]. This is widespread, especially in South and Eastern Africa, Saudi Arabia, Yemen, and the western Indian Ocean. Recently, the virus has been reported from non-endemic regions due to the presence of varieties of hosts and vectors. Climate changing regimes, dynamic environmental factors, human and animal-movement as well as virus evolutionary factors have played a significant role in the inter-epizootic transmission and spread of viruses in non-endemic regions annually [33]. Based on the latest information, the last outbreak caused an economic loss of ~10M USD in Saudi Arabia and ~107M USD in Yemen [5, 40]. In Africa and Saudi Arabian countries, RVFV sero-prevalence in RVFV-related arboviruses ranges from 2.1- 9% in human [33,41]. The expanded occurrence of RVFV in new areas including non-endemic regions requires a more detailed understanding of the virus, so that a specific and reliable diagnosis and antiviral therapy can be designed and developed to control the virus and disease spread in new and non-endemic regions. The genome sequences of RVFV from both human and animal hosts have been submitted to GenBank by many research groups from different geographic regions collected during various periods. Currently, there is no WHO-approved vaccine or antiviral therapies available against RVFV. The expansion of RVFV in the Arabian Peninsula and non-endemic regions has raised an alarming situation and attracted the global researchers to perform a detailed genome analysis so that any possible emergence and spread of new strains in the regions or non-endemic areas can be identified. Currently, there is a lack of RVFV prevalence and status of new infections in both humans and livestock. So, there is an urgent need to perform a detailed prevalence study by collecting and analyzing the viral genome sequence analysis. In this work, we have successfully cloned, sequenced, and analyzed the RVFV-N gene from Saudi Arabia. The sequences showed high similarities with earlier deposited RVFV sequences from KSA. The multiple sequence alignment of the RVFV-N gene shows a total of 51 nucleotides and 31 amino acid sequence variations compared with other RVFV isolates from various hosts and geographical regions. The phylogenetic tree analysis formed a closed cluster with RVFV isolates (human and animal) reported from KSA. Additionally, there is some separate clustering observed with other isolates reported from different geographic regions and hosts. Our findings from this study are supported by the earlier reports [42-43]. These findings based on only the RVFV-N gene show that the circulating isolate is more like previously reported isolates from KSA. However, full-genome sequence analysis is urgently needed which will provide an opportunity to identify the emergence of any suspected recombinant strain/isolate with extended properties of the RVFV in this region or other non-endemic regions. Therefore, a continuous monitoring and detailed genome sequence analysis study is urgently required by performing both seroprevalences and molecular studies in the Kingdom.

The host factors play an important role in RVFV infection and virulence. Recently, in a study, approximately 900 genes with potential involvement in RVFV infection and replication were identified [44]. The RVFV has a unique feature of reassortment capacity with other closely related viruses and strains especially with co-circulating bunyavirus in the field. An example of the detection of the Nigari virus was during the RVFV outbreak in Mauritania in 2010 with possible co-infection in goats in that region [33-45]. Mutations in the NSs gene may allow the induction of innate pro-inflammatory immune responses and lead to attenuation of the virus [8]. The RVFV P78 protein is known as a membrane glycoprotein and plays a significant role in virus dissemination in mosquitoes, but in humans, their biological role is unknown. The genetic changes lead to high-level expression of P78 that may be used as a novel strategy for the attenuation of RVFV virulence and generation of safe RVFV vaccines [46].The diagnosis of RVFV is being performed by using both serological and molecular assays in both livestock, human and non-human primates- chimpanzees [32,33,41, 43,47,48, 49,50]. In earlier published reports the full-length coding sequence of RVFV-N, NSs, NSm, Gc, has been expressed in a baculovirus system and vaccinated in sheep and the Immunoreactivity profiles of the recombinant proteins in western blot and in indirect enzyme-linked immunosorbent assay have been evaluated [51]. In another study, a simple ELISA assay was developed to distinguish naturally infected animals from ones that have been vaccinated with a mutant virus by cloning, expression, and purification of two viral proteins namely N and NSs proteins [52]. Additionally, the RVFV-N protein was expressed in Escherichia coli (*E. coli*) and purified by histidine-tag-based affinity chromatography and rRVFV-N protein-based-IgG sandwich ELISA and IgM capture ELISA for human sera were established from Japan [47]. The diagnostic performance of an ELISA based on RVFV-recombinant nucleocapsid protein was validated for the detection of the IgG antibody in livestock and Domestic Ruminants and showed the high diagnostic accuracy of the RVFV rNP I-ELISA [48]. Recently, the screening of anti-RVFV IgG and IgM was performed in stored serum samples from people with arbovirus symptoms in Italy, and 10% of samples found positive [33]. It has been observed in the past few decades the frequent reemergence of RVFV in Africa and the Arabian Peninsula. A systemic analysis of seroprevalence study reported 80% RVFV in all five African countries [5,6]. RVFV continues to spread to parts of Africa where it was not previously detected, and the introduction of the disease into other countries remains a potential threat [3].

The overall information generated from this study is very valuable to identify the emerging virus strain as we have analysed the N gene sequence with earlier reported isolates starting from 1976-2020 and both nucleotide and amino acids variation were identified. Based on these findings, it is expected that there will be more genetic variations in the other part of the viral genome that may facilitate the emergence of new virus strain not only in the Kingdom but also in other non-endemic regions. These possibilities raised a serious concern to urgently perform a detailed study based on the full genome sequence analysis, so that not only an early detection and continuous surveillance system but also a disease control strategy as well as an effective and affordable vaccine can be designed and developed for the protection of human and livestock in the Kingdom as well as non-endemic regions.

## Conclusion:

We report the cloning, sequencing, and phylogenetic analysis of the RVFV-N gene from KSA. This information will be further used for full genome sequence analysis as well as routine screening of RVFV prevalence by developing a specific and sensitive in-house serological and molecular assay in KSA. A continuous surveillance system will be highly useful to develop an effective control strategy of RVFV in non-endemic areas. This developed technology will also be used to carry out the RVFV testing of travellers and introduced livestock, mainly from Africa and other countries in the Kingdom.

## Funding:

The authors acknowledge the generous charitable donation from the Late Sheikh Ibraheem Ahmed Azhar in the form of reagents and supplies as a contribution to the scientific research community.

## Author contributions:

AMH: Sample collection and processing. AAZ: Methodology, validation, data curation; writing, and editing. AAA: Review and editing. SSS: Writing-original draft and final editing, software, validation, data curation, and formal analysis. EIA: Supervision, project administration; investigation, resources, funding acquisition.

## Figures and Tables

**Table 1:** Sequence identity matrix for RVFV-SIAU-N gene with selected isolates

**S. No**	**Accession Nos.**	**Abbreviations**	**Isolated Year**	**Locations**	**Hosts**	**% Identity (NT)**	**% Identity (AA)**
1	MG659986	RVFV	1976	Zimbabwe	Human	98.6	97.1
2	EU312125	RVFV	1985	Angola	Human	98.5	96.7
3	JF311390	RVFV	1991	Madagascar	Human	99.1	97.9
4	EU312106	RVFV	1991	Madagascar	Bovine	99.1	97.9
5	JF311391	RVFV	1991	Madagascar	Bovine	99	97.5
6	EU312108	RVFV	1991	Madagascar	Bovine	99	97.5
7	EU312107	RVFV	1991	Madagascar	Bovine	98.9	97.9
8	EU312128	RVFV	1997	Kenya	Human	99	97.5
9	KU978774	RVFV	1998	Kenya	Human	99.1	97.9
10	DQ380169	RVFV	1998	Kenya	Human	99.1	97.9
11	EU312126	RVFV	1998	Somalia	Caprine	98.9	97.5
12	KY196511	RVFV	1999	South Africa	buffalo	98.6	96.7
13	EU312127	RVFV	1999	South Africa	Buffalo	98.5	96.3
14	KU978775	RVFV	2000	Saudi Arabia	Human	99.5	98.7
15	DQ380170	RVFV	2000	Saudi Arabia	Human	99.4	98.3
16	KX096943	RVFV	2001	Saudi Arabia	Aedes.arabiensis	99.3	98.3
17	KX096940	RVFV	2006	Kenya	Aedes ochraceus	98.5	96.7
18	JQ840746	RVFV	2007	Sudan	Human	98.6	96.7
19	JQ820482	RVFV	2007	Sudan	Human	98.6	96.7
20	KX944825	RVFV	2008	South Africa	Bovine	98.5	96.7
21	KX944824	RVFV	2008	South Africa	Bovine	98.5	96.7
22	KX944823	RVFV	2008	South Africa	Bovine	98.5	96.7
23	KX944822	RVFV	2009	South Africa	Bovine	98.5	96.7
24	JQ820476	RVFV	2010	Sudan	Human	98.9	97.1
25	JQ820481	RVFV	2010	Sudan	Human	98.9	97.1
26	JQ820477	RVFV	2010	Sudan	Human	98.7	96.7
27	JQ820475	RVFV	2010	Sudan	Human	98.6	96.7
28	JQ820473	RVFV	2010	Sudan	Human	98.6	96.7
29	MT561462	RVFV	2010	Sudan	Human	98.7	96.7
30	MG972974	RVFV	2011	Namibia	Springbok	98.6	97.1
31	ON060812	RVFV	2018	Uganda	Human	98.6	96.7
32	ON060798	RVFV	2018	Uganda	Human	98.6	97.1
33	ON060792	RVFV	2018	Uganda	Human	98.6	97.1
34	ON060783	RVFV	2018	Uganda	Human	98.6	97.1
35	ON060780	RVFV	2018	Uganda	Human	98.6	97.1
36	ON060821	RVFV	2018	Uganda	Human	98.5	96.3
37	ON060810	RVFV	2018	Uganda	Human	98.5	96.7
38	ON060774	RVFV	2019	Uganda	Human	98.6	97.1
39	ON060833	RVFV	2020	Uganda	Human	98.6	97.1
